# Morpholino-Mediated Knockdown of Ciliary Genes in *Euplotes vannus*, a Novel Marine Ciliated Model Organism

**DOI:** 10.3389/fmicb.2020.549781

**Published:** 2020-10-19

**Authors:** Danxu Tang, Xiaoyu Wang, Jingyi Dong, Yuan Li, Feng Gao, Haibo Xie, Chengtian Zhao

**Affiliations:** ^1^Institute of Evolution & Marine Biodiversity, Ocean University of China, Qingdao, China; ^2^Laboratory for Marine Biology and Biotechnology, Qingdao National Laboratory for Marine Science and Technology, Qingdao, China; ^3^Ministry of Education Key Laboratory of Marine Genetics and Breeding, College of Marine Life Sciences, Ocean University of China, Qingdao, China

**Keywords:** *Euplotes vannus*, cilia, C21ORF59, morpholino, gene knockdown, ZMYND10

## Abstract

Cilia are highly conserved organelles present in almost all types of eukaryotic cells, and defects in cilia structure and/or function are related to many human genetic disorders. Single-celled ciliated protists, which possess diverse types of cilia, are remarkable model organisms for studying cilia structures and functions. *Euplotes vannus* is a representative ciliate with many intriguing features; for example, it possesses extensively fragmented somatic genomes and a high frequency of + 1 programmed ribosomal frameshifting. However, the molecular mechanisms underlying these remarkable traits remain largely unknown, mainly due to the lack of efficient genetic manipulation tools. Here, we describe the first application of a morpholino-based strategy to knockdown gene expression in *E. vannus.* Through interfering with the function of two ciliary genes, *ZMYND10* and *C21ORF59*, we showed that these two genes are essential for the ciliary motility and proliferation of *E. vannus* cells. Strikingly, both ZMYND10- and C21ORF59-knockdown cells developed shorter cilia in the ventral cirri, a special type of ciliary tuft, suggesting a novel role for these genes in the regulation of cilia length. Our data provide a new method to explore gene function in *E. vannus*, which may help us to understand the functions of evolutionarily conserved cilia-related genes as well as other biological processes in this intriguing model.

## Introduction

Cilia are highly conserved, complex, centriole-derived, microtubule-containing organelles that are found in almost all types of eukaryotic cells (Satir and Christensen, 2007). According to their movement capability, cilia are usually classified as either motile or primary (immotile) cilia. Motile cilia are essential for cellular movement or surface fluid flow, and primary cilia mainly perform sensory and signaling functions. In motile cilia, the microtubule composition is generally a “9 + 2” configuration, with nine doublet microtubules surrounding two central singlets, while in primary cilia, the composition is a “9 + 0” configuration that is missing the central singlets (Song et al., 2016).

Defects in cilia structure and/or function are related to many human genetic disorders, collectively termed as ciliopathies (Hildebrandt et al., 2011). For instance, ciliary motility defects lead to primary ciliary dyskinesia (PCD), an inherited disease that can cause chronic respiratory infections, visceral translocation, and infertility in humans (Hildebrandt et al., 2011; Song et al., 2016). The structure and function of cilia are relatively difficult to study in humans or other mammals, thus our current understanding of the mechanisms of ciliary formation is mainly based on a model organism, the green algae *Chlamydomonas reinhardtii*, which contains only two equal-length flagella. In contrast to *C. reinhardtii*, ciliated protists possess diverse types of cilia, and thus could be remarkable model organisms for studying ciliogenesis, especially for the study of multicilia, which are essential for body fluid flow during vertebrate embryonic development (Brooks and Wallingford, 2014).

Ciliates are among the most evolved and complex single-celled eukaryotes. They are found in diverse habitats across the globe and were the first observed and described eukaryotic microorganisms, as their study dates back to the late 17th century (Clamp and Lynn, 2017). In addition to the presence of cilia in at least one of their life stages, ciliates are also characterized by their nuclear dimorphism (i.e., possessing both a somatic macronucleus and germline micronucleus within each cell) and a special sexual process called conjugation (Jiang et al., 2019; Gong et al., 2020). Due to these unique characteristics, ciliates, such as *Paramecium aurelia* and *Tetrahymena thermophila*, have been widely used as research models (Singh et al., 2014; Chen et al., 2019; Wang et al., 2019).

Euplotid ciliates, represented by *Euplotes vannus*, the focus of this work, are the most highly differentiated and complicated group of ciliates. Euplotid cells differentiate into dorsal-ventral sides, and their cilia are diversified into different types, including cirri on the ventral side, which are composed of dozens or 100s of cilia joined together to form a tuft that functions as a “leg”; adoral zone membranelles, which are composed of rows of cilia that are fused together to form multiple membranelles, whose constant beating are vital for food absorption and function as a “mouth”; and single shorter cilia on the dorsal side, which are arranged in multiple rows and may possess sensory functions (Lynn, 2008; Jiang et al., 2010; Kloetzel and Brann, 2012). In addition, euplotids have many other intriguing and unique biological features. For example, they have extensively fragmented somatic genomes; the whole macronuclear genome of *E. vannus* contains more than 25,000 complete nanochromosomes, with an average size of 1.5 kb (Chen et al., 2019). Euplotids also have a high frequency of + 1 programmed ribosomal frameshifting (Wang et al., 2016; Lobanov et al., 2017; Chen et al., 2019), stop codon reassignment (Swart et al., 2016), and strong resistance to environmental stressors (Kim et al., 2018). However, the molecular mechanisms underlying these remarkable traits remain largely unknown.

Although the macronuclear genome of *E. vannus* has been released, tools for efficient genetic manipulation are still unavailable. *Euplotes* have been shown to contain more than 1,000 copies of their nanochromosomes (Baird and Klobutcher, 1991), meaning that there are more than 1,000 copies of most genes in the macronucleus, which hinders the use of conventional genetic manipulation methods, including CRISPR/Cas9 and TALEN gene editing techniques. Here, we performed gene silencing experiments using RNAi and antisense oligonucleotide methods. We showed that gene silencing can be achieved in *E. vannus* via microinjection of morpholino antisense oligonucleotides. Knockdown of *ZMYND10* or *C21ORF59*, two ciliary genes related to human PCD (Austin-Tse et al., 2013; Moore et al., 2013; Zariwala et al., 2013), resulted in cilia motility defects, suggesting that the roles of these ciliary motility proteins are conserved from single-celled organisms to metazoans. Unexpectedly, length of the cirri was significantly shorter in the knockdown cells, providing the first evidence that these proteins may regulate both ciliary motility and length control in *E. vannus*. Our data suggest that gene silencing in *E. vannus* can be achieved using morpholino antisense oligonucleotides, which may help us to explore the functions of evolutionarily conserved cilia-related genes as well as other intriguing fundamental biological processes in *E. vannus.*

## Results

### Cilia Diversity in *Euplotes vannus*

**FIGURE 1:**
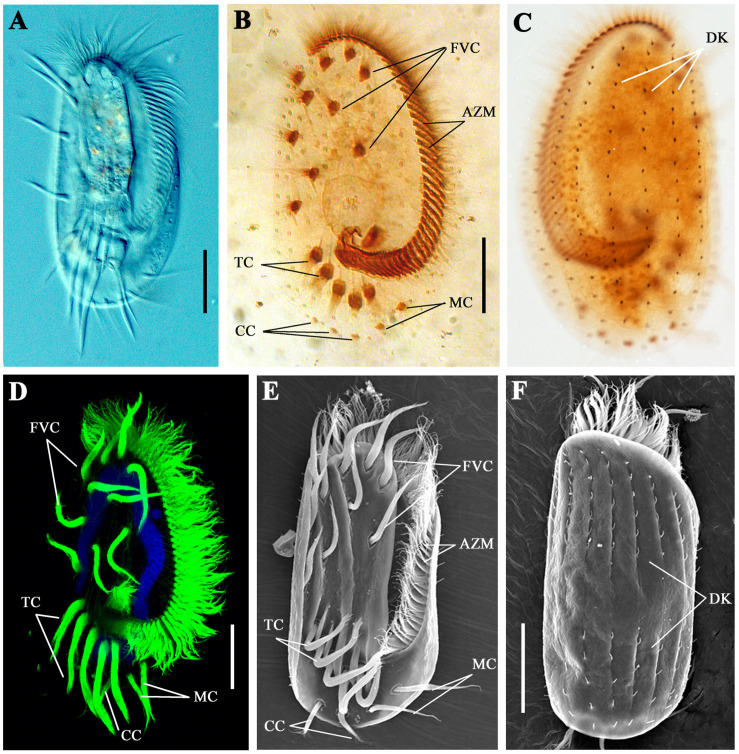
Cilia in *Euplotes vannus.* **(A)** DIC image showing the ventral side of a living *E. vannus* cell. Ventral **(B)** or dorsal **(C)** views of *E. vannus* after protargol staining showing the position of different cilia. **(D)** Confocal image showing cilia visualized with anti-acetylated tubulin antibody. Nucleus was labeled with DAPI in blue. **(E)** Ventral view of a cell using scanning electron microscopy (SEM). **(F)** Dorsal view of *E. vannus* showing the position of dorsal kineties. Arrows point to different type of cilia. AZM, adoral zone membranelles; CC, caudal cirri; DK, dorsal kineties; FVC, frontoventral cirri; MC, marginal cirri; TC, transverse cirri. Scale bars: 20 μm.

Cilia are essential for the movement and feeding behavior of ciliates. As one of the most complicated single-celled organisms, *E. vannus* has evolved highly diverse types of cilia (Figures 1A–F). The adoral zone membranelles (AZM), the most important component of the oral apparatus, are formed by fusion of multiple cilia in each membranelle, and their continuous beating is important for the ingestion of food into the “mouth” (Figure 1B). From the ventral view, 10 frontoventral cirri (FVC), 2 marginal cirri (MC), 2–3 caudal cirri (CC), and 5 transverse cirri (TC) were present in each cell (Figures 1B,D,E). The coordinate movement of these cirri is crucial for the swimming of *E. vannus* as well as its crawling behavior on different substrates. Moreover, single short cilia were aligned in multiple rows to form dorsal kineties (DK) (Figures 1C,F), which are immotile and may perform sensory functions (Lynn, 2008).

### Knockdown of Ciliary Genes in *Euplotes vannus* by RNAi

In reverse genetics, RNA interference (RNAi) is an outstanding method for knocking down specific genes and has been widely used in many model organisms, including *Paramecium*, *Trypanosomes*, *Planaria*, *Caenorhabditis elegans*, and *Drosophila*. Therefore, we first tested the possibility of disrupting gene function using an RNAi method. Considering that cilia are one of the most prominent features of *E. vannus*, we selected *ZMYND10* and *C21ORF59*, two genes required for ciliary motility, to test whether knockdown of these genes would affect cilia development. Sequence comparison showed that these two proteins are highly conserved from protists to humans (Supplementary Figures S1, S2).

**FIGURE 2:**
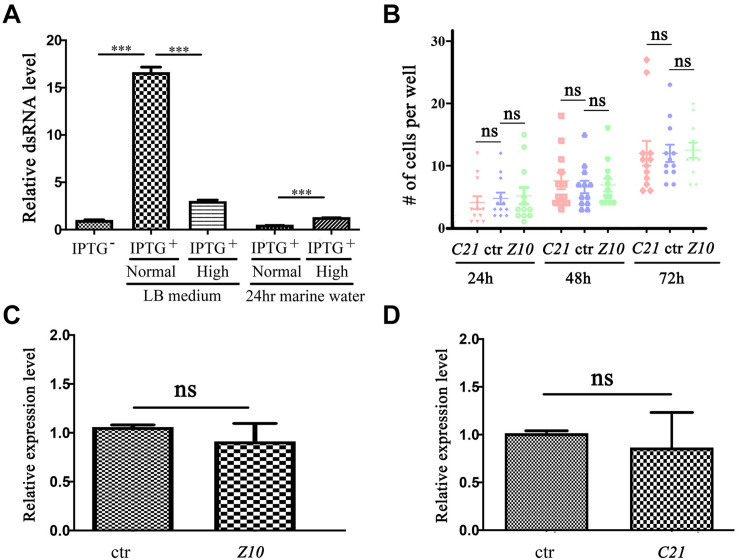
RNA interference in *Euplotes vannus.* **(A)** Bar graph showing relative expression level of *ZMYND10* dsRNA after IPTG induction at different conditions as indicated. The second and third bars show dsRNA expression after IPTG induction at normal or high salinity LB medium. The fourth and fifth bars show dsRNA expression in bacteria after 24-h incubation in marine water. These bacteria were induced and precultured in normal or high salinity LB medium before treatment. **(B)** Dot plot showing the number of cells per well after feeding with RNAi-expressing bacteria at different time points as indicated. **(C,D)** qPCR results showing the expression of *ZMYND10* (*Z10*) or *C21ORF59* (*C21*) in *E. vannus* fed with control or corresponding dsRNA-expressing bacteria as indicated. These bacteria were induced at normal LB culture medium. ^***^P < 0.001.

We cloned the partial cDNA of these genes into the pL4440 RNAi feeding vector and transformed these constructs into *Escherichia coli* (*E. coli*) strain HT115 (DE3). Considering that *E. vannus* is a marine ciliate that needs to be cultured in seawater, we first examined the double-stranded RNA (dsRNA) induction efficiency in *E. coli* cultured in high-salinity LB medium. Compared with that in regular LB medium, the relative dsRNA expression level was significantly lower after IPTG induction in high-salinity LB medium (Figure 2A). During *E. vannus* culture, we added *E. coli* to marine water as a food source on a daily basis. We further tested the stability of dsRNA in marine water. After IPTG induction, we transferred *E. coli* cultured in regular or high-salinity LB medium to marine water. Twenty-four hours later, we found that dsRNA was almost undetectable for both conditions, although high salinity-cultured *E. coli* contained slightly more dsRNA (Figure 2A). These results were further confirmed with two additional interference fragments, which suggested that dsRNA induction and maintenance were compromised in a high-salinity environment.

We also tested whether the induced dsRNA from bacteria cultured in normal LB medium was sufficient to interfere with gene expression. We fed *E. vannus* with *ZMYND10* or *C21ORF59* dsRNA-expressing *E. coli* and checked the expression of the endogenous genes via qPCR. The feeding experiments were performed both in small-scale cultures in 24-well plates and large-scale feeding in flasks. Our results suggested that both conditions failed to inhibit either gene expression or the growth of *E. vannus* cells (Figures 2B–D and data not shown). In addition, we also performed RNAi experiments with several other genes related to ciliogenesis, including *IFT43*, *IFT52*, and *BBS7*. Again, cilia development appeared to be grossly normal in RNAi-fed *E. vannus* cells (data not shown).

Finally, we synthesized dsRNA *in vitro* and directly injected it into *E. vannus* cells. The results of qPCR analysis showed that the expression levels of the corresponding genes remained unchanged in the injected cells (Supplementary Figure S3). These results suggested that gene silencing using dsRNA interference may not be an efficient method in *E. vannus* cells.

### Knockdown of the Motile Cilia-Related Gene *ZMYND10* via Morpholino Oligonucleotides

**FIGURE 3:**
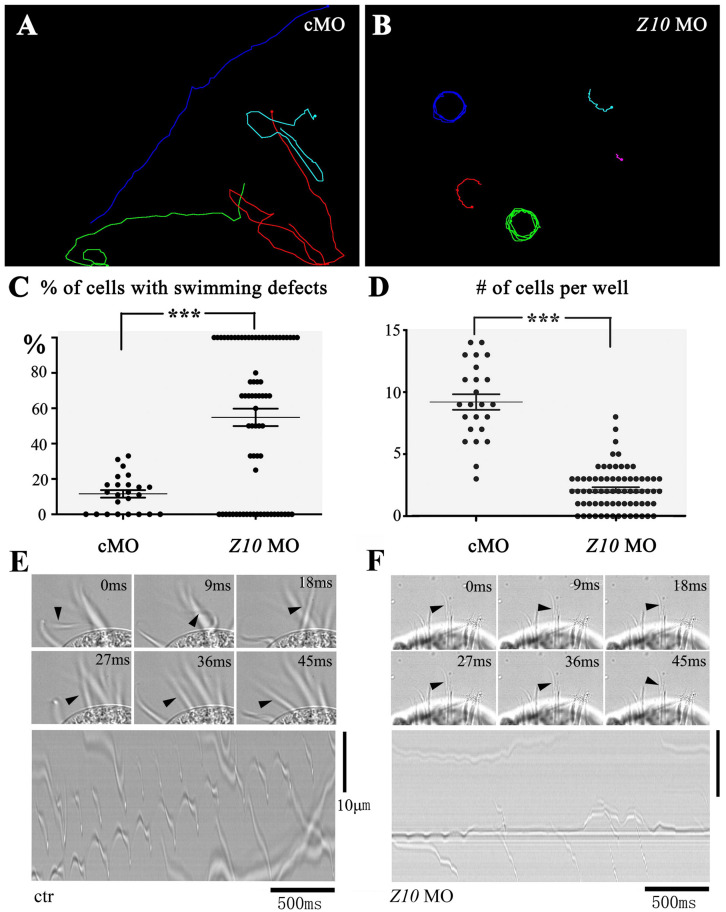
Swimming defects in ZMYND10-KD cells. Swimming paths of control **(A)** and ZMYND10-KD cells **(B)** as derived from Supplementary Movies S1 – S4. **(C)** Dot plot showing the percentages of cells with swimming defects. Each dot represents a group of cells proliferated from a single injected cell. **(D)** Plot showing the number of cells per well at 16-h after injection. Each dot represents the number of cells from a single injected cell. Still images showing the beating patterns of the transverse cirri in control **(E)** or *ZMYND10* MO **(F)** injected cells. Arrowheads indicate the position of an individual cirrus. Kymographs of the cirri beating were shown in the bottom. ^***^P < 0.001.

Morpholino antisense oligonucleotides (MOs) are nucleotide analogs that have been widely used to interrupt gene function through their binding activity either at the transcription start site or splice site of pre-mRNA to block translation or mRNA splicing (Summerton and Weller, 1997; Corey and Abrams, 2001). Next, we tested the possibility of knocking down ciliary genes using MOs. We first designed a MO to target *ZMYND10*. After the *ZMYND10* MO was microinjected into the cytoplasm of *E. vannus*, we observed that the majority of the progeny cells displayed severe swimming defects at 16 h after injection. In the control MO-injected group, all the cells could swim smoothly, with a linear swimming path (Figure 3A and Supplementary Movies S1, S2). In contrast, the *ZMYND10* MO-injected cells mostly showed a circular swimming path (Figures 3B,C and Supplementary Movies S3, S4). In addition, we compared the number of cells per well between the control and MO-injected groups. The number of *E. vannus* was significantly decreased in the *ZMYND10* MO-injected groups (Figure 3D). Morpholinos may cause off-target effects due to non-specific binding to other genes. Therefore, we designed a four-base mismatch MO that is similar to the experimental *ZMYND10* MO. Similar to the standard control MO, microinjection of the mismatch MO did not lead to any swimming defects. The proliferation of injected *E. vannus* was also normal (Supplementary Figure S4). Moreover, we examined the expression of *ZMYND10* in MO-injected cells and found no expression change of this gene, suggesting that *ZMYND10* MO blocked the translation of mRNA, but not affecting its expression level (Supplementary Figure S4F).

To further confirm these phenotypes, we examined whether overexpression of *E. vannus ZMYND10* mRNA could rescue the ciliary defects observed in ZMYND10-KD cells. We cloned the full-length *ZMYND10* gene into the pUCm-T vector, together with its 5′ and 3′ UTRs. The *ZMYND10* gene contains only one exon, and the encoded protein is highly conserved from ciliates to humans (Supplementary Figures S1, S5A,B). To circumvent potential translation blocking of the synthesized mRNA, we made seven synonymous mutations in the MO target site to prevent the MO from binding to the synthesized mRNA (Supplementary Figure S5C). When the *in vitro* transcribed *ZMYND10* mRNA was injected together with the MO, the swimming defects were substantially diminished compared with those in *E. vannus* injected with MO alone at 16 h after microinjection (Supplementary Figures S6A–C and Supplementary Movie S7, S8). The number of cells in each well was also significantly increased (Supplementary Figure S6D). These results indicated that the swimming and proliferation defects observed in ZMYND10-KD cells were due to the absence of ZMYND10 protein.

### Cilia Motility Was Compromised in *ZMYND10* MO-Injected Cells

The swimming of *E. vannus* depends on the coordinated beating of motile cilia, including both cilia in the AZM and multiple cirri on the ventral side. To better clarify the reason for the swimming defects in the knockdown cells, we examined the ciliary movement of these cells using a high-speed video microscope. In the control group, all the cirri beat coordinately to ensure its normal swimming behavior. We observed that the AZM cilia beat at a relatively high frequency (30 ± 1.8 Hz), while cirri moved at a low frequency with different beating pattern (Figure 3E and Supplementary Movie S5). This phenomenon prompted us to hypothesize that the AZM cilia not only assist in the ingestion of food into the cytostome, but also play a major role during cell movement, and that cirri may function as rudders to control swimming direction, especially those localized to the posterior region, CC and TC (Figure 1). When examining cilia beating in ZMYND10-knockdown (KD) cells, we found that the beating of AZM was discontinuous. In many cells, only some of the AZM cilia were beating, while those in the posterior part often stopped beating (Supplementary Movie S6). The motility defects of cirri were even more dramatic in that most cirri became paralyzed, and some beat only occasionally (Figure 3F and Supplementary Movie S6).

### Knockdown of *ZMYND10* Led to Disorganized Cirri in *E. vannus*

**FIGURE 4:**
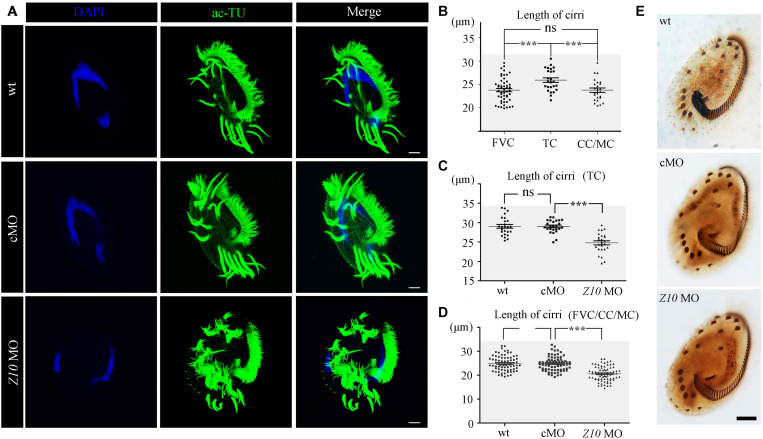
Cilia phenotype in ZMYND10-KD cells. **(A)** Confocal images showing cilia in wild type (wt), control MO (cMO) and *ZMYND10* MO injected cells as visualized with anti-acetylated tubulin (ac-TU) antibody (green). Nuclei were counterstained with DAPI in blue. **(B)** Statistical dot plot showing length of different types of cirri in wild-type *E. vannus* as indicated in Figure 1D. **(C)** Dot plot showing the length of transverse cirri in different group of cells as indicated. **(D)** Dot plot showing the length of cirri in different group of cells as indicated. **(E)** Silver staining results showing the distribution of AZM and cirri in different type of cells as indicated. Scale bars: 10 μm. ns, not significant; ***P < 0.001.

Next, we performed immunohistochemistry to investigate ciliogenesis using an anti-acetylated alpha tubulin antibody. The staining results suggested that cilia in the AZM and ventral cirri were preserved in ZMYND10-KD cells (Figure 4A). Notably, cirri displayed a brush-like structure, with multiple cilia sticking together to form a ciliary bundle in wild-type and control MO-injected cells. Interestingly, multiple individual cilia could be easily identified within each cirrus in the knockdown cells, suggesting that cilia were separated from each other in the absence of ZMYND10 proteins (Figure 4A). Such a phenotype was never observed in cells injected with the control or mismatch MO (Supplementary Figure S4). When comparing the length of different types of cirri in wild-type cells, we found the transverse cirri (TC) were much longer than the other types of cirri and the lengths of the FVC, CC, and MC were comparable to each other (Figure 4B). Strikingly, the lengths of all types of cirri were significantly shorter in ZMYND10-KD cells than in control cells (Figures 4C,D). Noticeably, both the bundle-like structure and length defects of cirri could be rescued by overexpression of *ZMYND10* mRNA (Supplementary Figures S6E,F). Finally, protargol-staining experiment results showed that the number and distribution of cirri, AZM, and dorsal kineties were similar in the knockdown and control groups, suggesting that ZMYND10 only affects the movement and length of cirri, but not their localization (Figure 4E).

**FIGURE 5:**
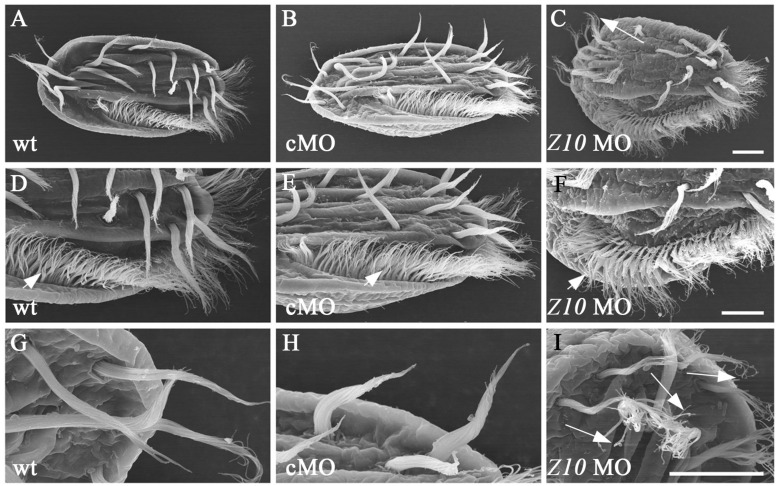
SEM analysis of cilia in ZMYND10-KD cells. **(A–I)** Scanning electron microscopy results showing the ultrastructure of cilia in cirri and AZM of wild type, control MO and *ZMYND10* MO injected cells. **(D–F)** Enlarged views showing AZM cilia (arrowheads). **(G–I)** Enlarged views showing the tip structure of FVC cilia. Arrows point to the dispersed cilia at the tip of cirri in a *ZMYND10* MO injected cell. Scale bars: 10 μm.

To compare the ultrastructural differences between control and ZMYND10-KD cells, we performed a scanning electron microscopy (SEM) analysis. In wild-type or control MO-injected cells, the cirri and AZM cilia could be easily distinguished (Figures 5A–I). In the high magnification views, all the cilia stuck together to form a brush-like cirri (Figures 5G,H). In contrast, the cirri were clearly shorter and more disorganized in the knockdown cells (Figures 5C,I). Moreover, each individual cilium was separate from each other in the cirri, especially in the tip region (Figure 5I). Interestingly, the length and number of AZM cilia appeared to be normal in the KD cells, suggesting that ZMYND10 mainly regulates cilia motility, but not the formation of these cilia.

### Ciliary Motility Defects in C21ORF59-KD Cells

**FIGURE 6:**
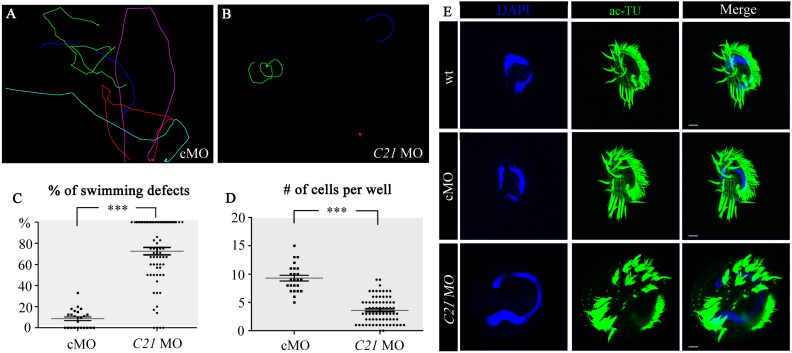
Cilia defects in C21ORF59-KD cells. **(A,B)** Swimming paths of cells injected with control MO (cMO) or *C21ORF59* (*C21*) MO. The corresponding movies were shown in Supplementary Movies S9, S10. **(C)** Dot plot showing percentages of cells with swimming defects in each well injected with cMO or *C21ORF59* MO. **(D)** Number of cells in each well injected with cMO or *C21ORF59* MO. **(E)** Confocal images showing cilia (green) in cMO or *C21ORF59* MO injected cells as indicated. Scale bars: 10 μm. ^***^P < 0.001.

Finally, we investigated the knockdown of another ciliary gene, *C21ORF59*, which is essential for the assembly of outer dynein arms in cilia, and hence regulates ciliary motility. Similar to ZMYND10-KD cells, *C21ORF59* MO also led to proliferation and swimming defects in *E. vannus* at 16 h after injection (Figures 6A–D and Supplementary Movie S9). High-speed video microscopy observations suggested that cilia were either completely paralyzed or showed reduced beating frequency in the KD cells (Supplementary Movie S10). The phenotype of dispersed cilia in the cirri was also present in C21ORF59-KD cells (Figure 6E and Supplementary Movie S10). Moreover, the length of the cirri was significantly shorter when *C21ORF59* was knocked down (Supplementary Figure S7A). Finally, we demonstrated that C21ORF59 was highly conserved and that cirri defects can also be rescued by microinjection of non-targeting *C21ORF59* mRNA (Supplementary Figures S7B–E, S8), further proving the specific phenotype of the morpholinos.

## Discussion

The study of protozoa has not stopped since the first protozoan was discovered by Antonie Van Leeuwenhoek 300 years ago. Ciliates are a large group of protozoans characterized by the presence of cilia that are essential for many biological functions. Ciliates are widely distributed worldwide and have evolved elaborate adaptive systems for extreme conditions (Hu et al., 2019). Among the various ciliates, species in the genus *Euplotes* are highly evolved and among the most complicated single-celled organisms. For example, *E. vannus* has evolved highly diverse cilia, which can either fuse together to form membranelles and function as a “mouth,” or form bristle-like appendages and function as a “leg” (Figure 1). These complex cilia structures ensure that *E. vannus* can perform several complicated movements in addition to swimming (e.g., walking, backward movement, and sharp turning). Moreover, their flexible genetic code system, together with its yet unknown mechanism of programmed ribosomal frameshifting, makes *E. vannus* one of the most intriguing model systems for many biological processes.

Although the genome of *E. vannus* was released recently (Chen et al., 2019), there are still no efficient tools for genetic studies. This is partly due to the presence of MAC nanochromosomes, which contain 100s to 1000s of copies of each gene, making regular genome editing methods (e.g., CRISPR/Cas9 and TALEN) unfeasible. RNAi, which is based on dsRNA-triggered post-transcriptional gene silencing, is a powerful tool for reverse genetics that has been widely used in many model systems, including *P. aurelia* and *C. reinhardtii*. In this study, we first tested the possibility of interrupting gene function by feeding *E. coli* cells containing an RNAi expression system to *E. vannus*. We designed DNA fragments against *ZMYND10*, *C21ORF59* and several other ciliary genes. All of these RNAi experiments failed to knockdown the expression of the target genes. The salinity may be one of the factors affecting RNAi efficiency in *E. vannus*, as both dsRNA induction and maintenance were compromised under high-salinity conditions. Nevertheless, this needs to be further confirmed by using another evolutionary related freshwater *Euplotes*.

On the other hand, *E. vannus* contains more than 1,000 copies of genes in the macronucleus, which may also hinder the use of regular RNAi methods. During RNAi feeding experiments, many *E. coli* cells died after added to the *E. vannus* culture medium. In such cases, *E. vannus* may not ingest enough *E. coli* cells nor the amount of dsRNA to trigger RNAi for such a large amount of mRNA. Interestingly, a recent study from another ciliate, *Oxytricha trifallax*, which contains a similar amount of nanochromosomes, suggested that small interfering RNAs (siRNAs) actually regulate the dose of DNA, that is, the chromosome copy number, but not mRNA silencing (Swart et al., 2013; Khurana et al., 2018). Moreover, mutation of *dcl-1*, the key gene involved in RNAi-mediated gene silencing, showed no evidence for mRNA expression changes, implying that there are different mechanisms in these ciliates compared to other model systems (Khurana et al., 2018). Altogether, these results suggested that RNAi may not be an efficient tool for gene silencing studies in *E. vannus.*

Alternately, we showed that gene silencing can be achieved in *E. vannus* via morpholino antisense oligonucleotides. MOs are synthetic antisense DNA analogs that can target a specific RNA *in vivo* to block mRNA translation or splicing. Both *ZMYND10* and *C21ORF59* are essential ciliary motility genes required for the assembly of dynein arms. Mutation of these two genes resulted in paralyzed cilia in both humans and zebrafish. In this study, we showed that knockdown of these two genes via MOs also led to ciliary motility defects in *E. vannus*, suggesting the highly conserved roles of these genes. The paralyzed cilia phenotype was not present in *E. vannus* injected with either the standard control or mismatch MOs, which confirmed the specificity of gene function interruption via MOs. Moreover, we showed that ciliary motility defects can be rescued by co-injection of the corresponding mRNA, further validating the function of these genes.

In *E. vannus*, cirri, the tufts of cilia on the ventral side, are formed by dozens of cilia assembled together that beat coordinately during swimming or movement. Both immunostaining and SEM showed that the cilia were separate from each other in the KD cells, especially at the tip of the cirri. Further ultrastructural analysis showed no evidence of structural defects at the base of cirri between control and KD cells (Supplementary Figure S9). These data imply that the synchronized beating of cirri is achieved by coordinated ciliary movement. In KD cells, defects in ciliary motility disturbed cilia movement, hence affecting the organization of the cilia. Unexpectedly, the length of cirri was significantly shorter in KD cells. Mutation of *ZMYND10* and *C21ORF59* in humans and zebrafish mainly affected cilia motility, while cilia were still formed (Austin-Tse et al., 2013; Zariwala et al., 2013; Zhang et al., 2018). The shorter cilia in these KD cells suggests that these genes are required for ciliary motility but may also participate in the control of cilia length, especially for the cilia in the cirri, a special type of cilia bundle that is unique to ciliates. Further mechanistic analysis is necessary to decipher the role of these genes during the development of cirri.

Overall, our study established a method to explore gene functions during vegetative reproduction of *E. vannus.* Compared to other methods, we showed that MO-based knockdown can be an efficient method for gene silencing due to its specificity and simplicity. Notably, MOs block the translation of mRNA, but do not change the sequence of genomic DNA; hence, the knockdown effects will be transient. Indeed, due to the dilution of the MOs after cell division, we found cilia motility defects in injected cells were gradually recovered from 24 h post-injection. Further genomic editing tools are necessary to investigate the long-term roles of specific genes.

## Materials and Methods

### *E. vannus* Cell Culture

The collection and identification of *E. vannus* were performed as described by Jiang et al. (2019). First, cells were separated into monoclones and cultured in small Petri dishes containing filtered, sterilized marine water at 25°C for 7–10 days, with *E. coli* added daily as the food source. After reaching the maximum density, the cells were transferred to flasks for large-scale culture.

### RNAi Methods

The partial coding regions of the macronuclear genes *ZMYND10*, *C21ORF59*, *IFT43*, *IFT52*, and *BBS7* were amplified from the *E. vannus* genome and cloned into the L4440 RNAi expression vector. The primers used to amplify these genes are listed in Supplementary Table S1. The cloned constructs were transformed into an RNase III-deficient strain of *E. coli* with an IPTG-inducible T7 polymerase gene, HT115(DE3).

The interference strains were first cultured in a small centrifuge tube in normal LB medium (10 g of tryptone, 5 g of yeast extract, and 10 g of sodium chloride per liter) or high-salinity LB medium (10 g of tryptone, 5 g of yeast extract, and 35 g of sodium chloride per liter) overnight at 37°C. The next day, the bacteria were transferred to LB medium (normal or high salinity) at a volume ratio of 1:100 and cultured until the OD reached 0.5. Then, IPTG was added at a final concentration of 0.4 mmol/L. The bacteria were further cultured until the OD reached 1.0.

To quantitate dsRNA expression levels after induction, total RNA was isolated from IPTG-induced bacteria using the standard TRIzol extraction protocol. Reverse transcription was performed using HiScript III RT SuperMix (Vazyme). The qPCR reaction was set up using ChamQ Universal SYBR qPCR Master Mix (Vazyme Q711-02) and performed on an Applied Biosystems 7500 Fast Real-Time PCR system, with *recA* as an internal reference gene. The primers used for qPCR are listed in Supplementary Table S1.

To test the stability of dsRNA in seawater, 5 mL of IPTG-induced *E. coli* cultured in regular or high-salinity LB medium was first centrifuged at 7000 rpm for 3 min, and then the cell pellet was resuspended in 15 mL of seawater. Twenty-four hours later, the remaining bacteria were collected, and dsRNA expression levels were quantitated by qPCR.

For the bacterial feeding experiments, we first attempted small-scale cultures in 24-well plates. We put a single cell into each well and fed them dsRNA-expressing bacteria. Control *E. vannus* cells were fed the HT115 strain containing the L4440 empty vector. To feed *E. vannus* cells, a 1 mL aliquot of IPTG-induced bacteria (OD = 1.0) was collected, centrifuged, and resuspended in 12 mL of seawater. Then, 1 mL of the resuspended liquid was added to each well as a daily nutrient source until analysis. For the large-scale culture, 100 mL of seawater containing about 5 × 10^4^ *E. vannus* cells were cultured in a 500 mL flask, and 1 mL of IPTG-induced bacteria were added every day. We collected *E. vannus* cells after 7 days of feeding. Total RNA extraction and qPCR experiments were performed as previously described. The 18S rRNA gene of *E. vannus* was selected as a reference gene to evaluate the expression of endogenous genes.

For dsRNA injection, both sense and antisense RNA fragments were synthesized from linearized L4440 vectors using the MEGAscript T7 Transcription Kit (Thermo Fisher). Equal volumes of sense and antisense RNA (approximately 1 μg/μL each) were mixed together and microinjected into the cytoplasm of *E. vannus* cells. Approximately 30 cells were injected with each dsRNA mix. The injected cells were transferred back to seawater, and total RNA was isolated 24 h later to evaluate the expression of target genes.

### Morpholino, mRNA Preparation, and Microinjection

Morpholino antisense oligonucleotides were purchased from Gene Tools, LLC and dissolved in 60 μL of double distilled water to a final concentration of 40 μg/μL. The sequences of the MOs were: *ZMYND10*, 5′-CGTCTACACCTAAATT GTCGTTCAT-3′; *ZMYND10* mismatch, 5′-CGTCTACAttTAA cTTaTCGTTCAT-3′; *C21ORF59*, 5′-TCTTTTTGAAATGAAT GAGTACCAT-3′; and standard control MO, 5′-CCTC TTACCTCAGTTACAATTTATA-3′. For the rescue experiments, the full-length sequences of the macronuclear *ZMYND10* and *C21ORF59* genes, containing both the 5′ and 3′ UTRs, were first amplified from the *E. vannus* genome and cloned into the pUCm-T vector. The primers used are listed in Supplementary Table S1. Seven silent mutations in the MO target region were introduced to prevent translation blocking of the synthesized mRNA by MO. The sequences of corresponding mutations are shown in Supplementary Figures S5, S8. The capped mRNA was synthesized using the Ambion mMESSAGE mMACHINE mRNA transcription synthesis kit and injected together with MO at a final concentration of 750 ng/μL.

For microinjection, *E. vannus* cells were first incubated in a buffer solution (0.2% BSA in seawater) for 30 min. Then, a droplet of medium containing a single cell was picked up and placed onto an adhesive slide. The droplet was covered with mineral oil to prevent evaporation. Microinjection was conducted using an Eppendorf TransferMan^®^ 4r on a Leica Microsystem scope, with the following settings, pi = 400–600 and ti = 0.2. After injection, each cell was put back into a 24-well plate containing culture medium. The cells were cultured with bacterial feeding until analysis.

### Cell Proliferation and Swimming Analyses

At 16 h after injection, the number of cells in each well was counted under a MOTIC SMZ-140 stereoscopic microscope. To track cell movement, *E. vannus* cells were recorded under a 10 × objective with a Leica DFC450 CCD camera. Trajectory tracking was performed using ImageJ software.

### High-Speed Video Microscopy

To record cilia movement, *E. vannus* cells were first washed with sterilized seawater and then placed on a cover slide with a droplet of seawater. Then, the cover slide was placed upside down on a depression slide. Cilia movement was captured with a high-speed camera (Motion-BLITZ EoSens mini1; Mikrotron) mounted on a Leica SP8 confocal microscope under a 100 × oil objective. Movies were captured at a rate of 500 fps, and playback was set at 25 fps. Kymographs were generated using Image J software.

### Immunofluorescence and Protargol Staining Assay

*E. vannus* cells were first transferred to a staining plate and permeabilized in PHEM buffer (60 mM PIPES, 25 mM HEPES, 10 mM EGTA, 2 mM MgCl_2_, pH 6.9) containing 1% Triton X-100 for 2 min and then fixed in 2% paraformaldehyde for 10 min. After washing twice with TBST/BSA (10 mM Tris, 0.15M NaCl, pH 7.4, containing 0.1% Tween-20 and 3% BSA), the cells were incubated with an anti-acetylated tubulin antibody (1:500; Sigma) diluted in TBST/BSA for 1 h at room temperature (RT). Next, the cells were briefly washed with TBST/BSA and then incubated with the secondary antibody for 30 min at RT. Finally, the nuclei were stained with DAPI for 10 min at RT. Images were captured under a Leica SP8 confocal microscope.

For protargol staining, *E. vannus* cells were fixed in Bouin solution (75 mL of picric acid saturated aqueous solution, 25 mL of formalin, and 5 mL of glacial acetic acid) for 5–10 min, and then washed 3–4 times with distilled water. The cells were bleached with 0.1% sodium hypochlorite until they became transparent and then washed twice with distilled water. Cells were further processed for protargol staining as previously described (Pan et al., 2013).

### Scanning Electron Microscopy

*Euplotes vannus* cells were fixed in 2.5% glutaraldehyde (dissolved in seawater) for 10 min at 4°C and then rinsed three times with sterilized seawater for 50–60 min. The cells were gradually dehydrated in a graded series of ethanol (30, 50, 70, 80, 90, 95, and 100% for 10 min each), and then transferred to a CO_2_ critical point dryer (Leica EM CPD300) for drying. The cells were coated with gold using an ion coater (Leica EM ACE600), and samples were observed and photographed under a Hitachi S-4800 SEM.

### Statistical Analysis

To evaluate swimming defects, 24-well plates were placed under a dissecting microscope at 16 h after microinjection. *E. vannus* cells were considered to be defective if they remained static for more than 3 s or swam circularly after tapping the plates. Swimming trajectories that were either a dot or circular after software analysis were considered defective cells, while trajectories that were linear were considered normal cells. The swimming defect ratio was determined as the percentage of defective cells in each well. Cirri length was measured in confocal images of *E. vannus* cells immunostained with an anti-acetylated tubulin antibody using Image J software. All experiments in this study were repeated at least three times. All statistical graphs and analyses were generated using Prism software. Scatterplot data are presented as mean ± SD, and statistical significance was evaluated using Student’s *t*-test for unpaired data. A *P*-value less than 0.05 was considered statistically significant, ^∗^*P < 0.05*, ^∗∗^*P* < 0.01, ^∗∗∗^*P* < 0.001.

## Data Availability Statement

The raw data supporting the conclusions of this article will be made available by the authors, without undue reservation.

## Author Contributions

CZ designed the project. DT performed the microinjection and immunostaining experiments. XW and DT performed the RNAi experiments. HX performed the high video recording experiments. YL carried out statistical analysis. JD and HX performed the SEM experiments. DT, FG, HX, and CZ analyzed the data and wrote the manuscript. All authors contributed to the article and approved the submitted version.

## Conflict of Interest

The authors declare that the research was conducted in the absence of any commercial or financial relationships that could be construed as a potential conflict of interest.

## Supplementary Material

Supplementary Figure 1 Alignment of amino acid residues of the ZMYND10 orthologs.

Supplementary Figure 2 Alignment of amino acid residues of the C21ORF59 orthologs.

Supplementary Figure 3 Microinjection of dsRNA in *E. vannus.* Bar graphs showing qPCR results of the expression of *ZMYND10* or *C21ORF59* genes in *E. vannus* injected with *in vitro* transcribed dsRNA.

Supplementary Figure 4 *ZMYND10* mismatch MO didn’t produce defects in *E. vannus.* **(A,B)** Swimming paths of cells injected with *ZMYND10* MO (*Z10* MO) or mismatch MO (MS MO). **(C)** Confocal images showing cilia (green) in *ZMYND10* MO or mismatch MO injected cells as indicated. **(D)** Number of cells in each well injected with *ZMYND10* MO or mismatch MO. **(E)** Dot plot showing percentages of cells with swimming defects in each well injected with *ZMYND10* MO or mismatch MO. **(F)** qPCR results showing the expression of *ZMYND10* mRNA in wild type, MS MO or *ZMYND10* MO injected cells at 16 h after injection. Scale bars: 10 μm.

Supplementary Figure 5 Sequences of *ZMYND10* in *E. vannus.* **(A)** Genomic sequence of the whole nanochromosome containing full length *ZMYND10* gene. Telomere sequences were highlighted in yellow. The open reading frame sequence was shown in upper case and the untranslated region (UTR) was shown in lower case. Underlined sequence shows the complementary binding site of *ZMYND10* MO. **(B)** Phylogenetic tree showing ZMYND10 protein in different species constructed by maximum likelihood methods. **(C)** Sequences showing MO targeting site of *ZMYND10* gene in wild type cells. The silent mutations were introduced (blue) to prevent MO binding in the synthesized sequence.

Supplementary Figure 6 Overexpression of *ZMYND10* mRNA rescued cilia defects in KD cells. Swimming paths of *E. vannus* cells injected with *ZMYND10* MO **(A)** or *ZMYND10* MO and *ZMYND10* mRNA **(B)**. The corresponding movies were shown in Supplementary Movies 7, 8. **(C)** Percentages of cells with swimming defects in different group of injected cells as indicated. **(D)** Dot plot showing number of cells per well at 16 h after injection in different groups as indicated. **(E)** Confocal images showing cilia (green) in ZMYND10-KD or rescued cells as indicated. **(F)** Dot plot showing length of cirri in ZMYND10-KD or rescued cells as indicated. Scale bars: 10 μm.

Supplementary Figure 7 Rescue of cilia defects in C21ORF59 KD cells by *C21ORF59* mRNA. **(A)** Length of cirri in different group of cells as indicated. **(B)** Length of cirri in C21ORF59-KD and rescued cells as indicated. **(C)** Confocal images showing cilia in control and rescued cells as indicated. **(D)** Number of cells per well in C21ORF59-KD and rescued cells as indicated. **(E)** Dot plot showing percentages of cells with swimming defects in C21ORF59-KD and rescued cells as indicated. C21: *C21ORF59* MO. Scale bar: 10 μm.

Supplementary Figure 8 Sequences of *C21ORF59* in *E. vannus.* **(A)** Genomic sequence of the whole nanochromosome containing full length *C21ORF59* gene. Sequences of telomere, UTR and MO binding sites were similar labeled as in Supplementary Figure 5. **(B)** Phylogenetic tree showing C21ORF59 protein in different species constructed by maximum likelihood methods. **(C)** Sequences of MO targeting site and corresponding silent mutations used for rescue experiments.

Supplementary Figure 9 Ultrastructural analysis of cirri in C21ORF59 KD cells. **(A–D)** SEM images showing the base of FVC cirri in control and ZMYND10 KD cells as indicated. Scale bars: 1 μm in **(A,C)** and 150 nm in **(B,D)**.

Supplementary Table 1 List of primers used in this study.

Supplementary Movie 1 Movie showing the swimming of *E. vannus* cells injected with control MO.

Supplementary Movie 2 Tracking of the swimming path of *E. vannus* cells injected with control MO.

Supplementary Movie 3 Movie showing the swimming of *E. vannus* cells injected with *ZMYND10* MO.

Supplementary Movie 4 Tracking of the swimming path of *E. vannus* cells injected with *ZMYND10* MO.

Supplementary Movie 5 High speed video showing cilia beating of *E. vannus* cells injected with control MO.

Supplementary Movie 6 High speed video showing cilia beating of *E. vannus* cells injected with *ZMYND10* MO. Video was recorded at 500fps and playback was set as 15fps.

Supplementary Movie 7 Movie showing the swimming of *E. vannus* cells injected with *ZMYND10* MO alone.

Supplementary Movie 8 Movie showing the swimming of *E. vannus* cells injected with *ZMYND10* MO and *ZMYND10* mRNA.

Supplementary Movie 9 Movie showing the swimming of *E. vannus* cells injected with *C21ORF59* MO.

Supplementary Movie 10 High speed video showing cilia beating of *E. vannus* cells injected with *C21ORF59* MO. Video was recorded at 500fps and playback was set as 15fps.
