# Supplementary material for: Morpholino-Mediated Knockdown of Ciliary Genes in Euplotes vannus, a Novel Marine Ciliated Model Organism
Source: Front Microbiol. 2020 Oct 19;11:549781. doi: 10.3389/fmicb.2020.549781 (PMC7604394; doi:10.3389/fmicb.2020.549781)
Supplement: Supplementary Figure 1 — Alignment of amino acid residues of the ZMYND10 orthologs. [file Data_Sheet_2.PDF]

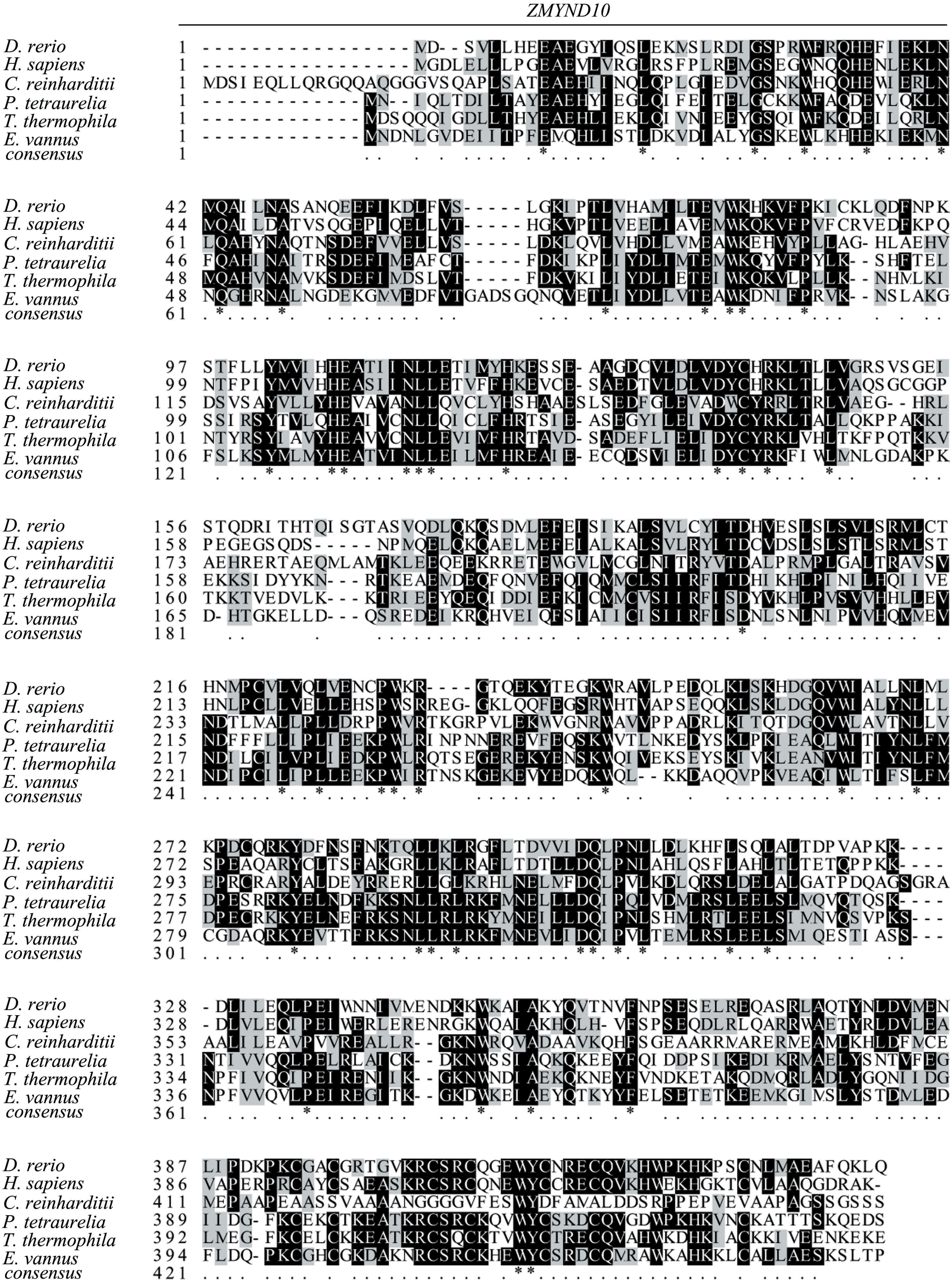
ZMYND10
D. rerio
H. sapiens
C. reinharditii
P. tetraurelia
T. thermophila
E. vannus
consensus

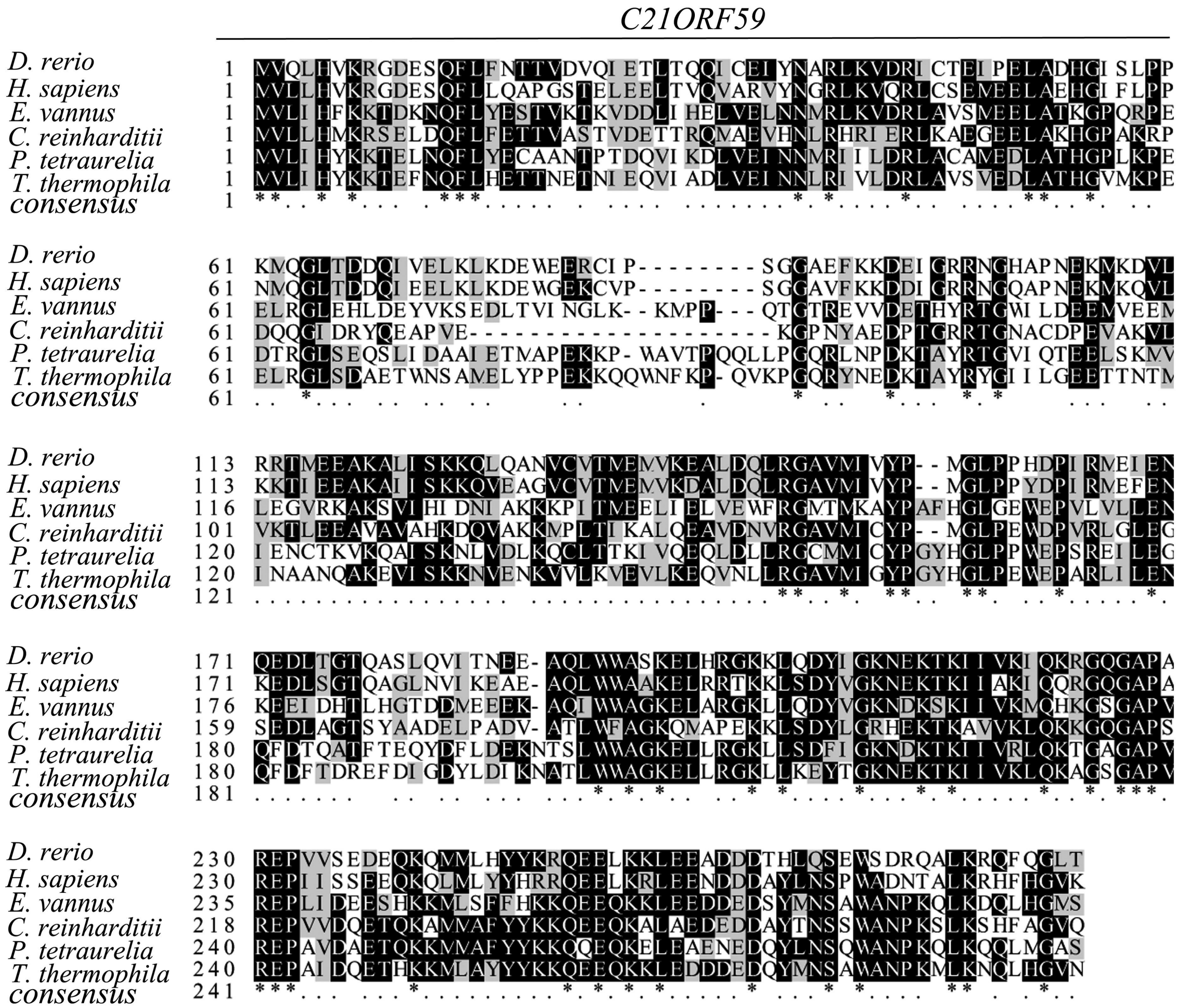

C21ORF59
D. rerio
H. sapiens
E. vannus
C. reinharditii
P. tetraurelia
T. thermophila
consensus

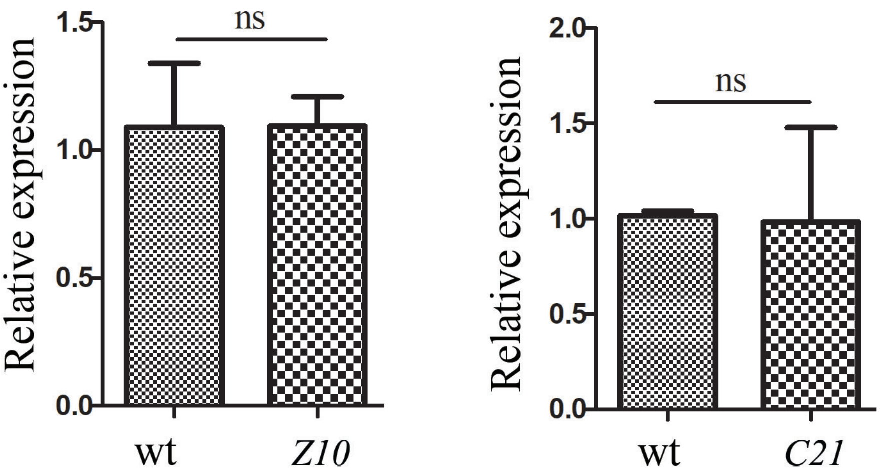
1.5
1.0
0.5
0.0
Relative expression
ns
wt
Z10
2.0
1.5
1.0
0.5
0.0
Relative expression
ns
wt
C21

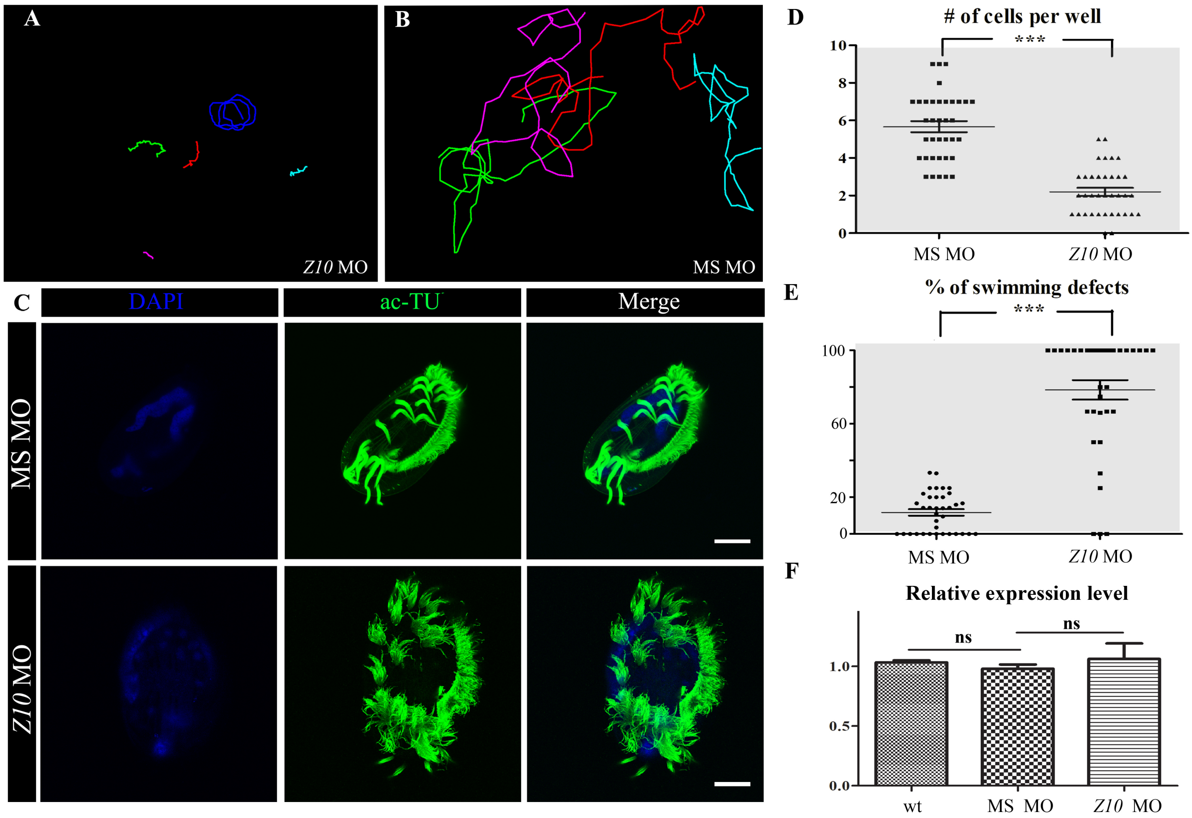
A
Z10 MO
B
MS MO
C
DAPI
ac-TU
Merge
MS MO
Z10 MO
D
# of cells per well
***
0
2
4
6
8
10
MS MO
Z10 MO
E
% of swimming defects
***
0
20
60
100
MS MO
Z10 MO
F
Relative expression level
ns
ns
0.0
0.5
1.0
wt
MS MO
Z10 MO

A

```
   1 ccccaaaaccccaaaaccccaaaaccccaaaaccccgtgtataaatttgg
  51 aatttgagataaatattttaaatattcccggattaaagATGAACGACAAT
 101 TTAGGTGTAGACGAGATAATTACCCCATTCGAAATGCAACATCTCATTAG
 151 CACACTTGATAAGGTAGATATTGCCCTCTATGGCTCGAAGGAATGGCTCA
 201 AGCATCATGAAAAGATTGAGAAAATGAACAACCAAGGCCATAGAAATGCT
 251 CTAAACGGGGATGAGAAAGGAATGGTCGAAGATTTTGTTACAGGAGCTGA
 301 TTCAGGTCAAAATCAAGTAGAAACTCTAATTTATGACCTTCTGGTCACAG
 351 AAGCTTGGAAGGATAATATCTTCCCGCGTGTTAAAAACAGTCTTGCCAAA
 401 GGATTCTCGCTTAAATCTTATATGTTGATGTACCATGAAGCTACAGTGAT
 451 TAATCTTTTGGAAATCCTTATGTTCCACAGAGAGGCTATCGAGGAGTGCC
 501 AGGATAGTGTCATCGAACTCATAGATTATTGTTACAGAAAATTCATATGG
 551 CTCATGAACCTGGGAGATGCAAAGCCAAAAGATCATACAGGAAAGGAACT
 601 GCTCGATCAATCTAGAGAAGATGAGATTAAAAGACAGCATGTTGAGATCC
 651 AATTTTCAATTGCGATAATTTGAATTTCTATTATTAGGTTTATCAGTGAT
 701 AATCTATCGAACCTGAACATCCCGGTCGTCCATCAGATGATGGAAGTTAA
 751 TGACATCCCATGTATCCTCATTCCTCTGCTAGAAGAGAAGCCATGGATTA
 801 GGACTAACTCTAAAGGAGAAAAGGAAGTATATGAGGATCAGAAGTGGCAA
 851 CTCAAGAAAGACGCCCAGCAAGTACCTAAAGTCGAAGCCCAGATTTGGCT
 901 GACTATCTTCAGTCTTTTCATGTGCGGGGACGCACAGAGAAAGTATGAAG
 951 TGACAACATTTAGAAAATCAAACCTTTTACGTTTGAGGAAGTTTATGAAT
1001 GAAGTTCTCATTGATCAGATTCCTGTCTTGACAGAAATGCTAAGATCTCT
1051 AGAGGAGCTCTCCATGATTCAGGAGTCCACCATTGCATCCTCAAATCCCT
1101 TTGTAGTTCAGGTTCTCCCAGAGATTAGAGAAGGAATTACCAAGGGTAAG
1151 GATTGGAAGGAAATAGCAGAGTATCAGACGAAGTATTACTTTGAACTCTC
1201 AGAGACAGAGACTAAAGAGGAAATGAAGGGTATCATGTCCCTCTACAGCA
1251 CGGATATGCTCGAGGACTTCCTTGATCAACCCAAATGCGGCCATTGAGGT
1301 AAAGACGCTAAGAATAGATGCAGTAGATGTAAGCATGAGTGGTACTGCAG
1351 CAGGGACTGACAGATGAGAGCATGGAAAGCTCACAAGAAGTTGTGAGCCT
1401 TACTTGCTGAAAGTAAGTCATTGACACCTTAAgagttccgtaaaagaaag
1451 agagctttgatttgatgaagccatagttttgcacgttgtcataaatatac
1501 taaatttatatatttaggcattaagaatgatgaaaatagtacaaagaagg
1551 ataaaaagaagcaggagaaaactgagtctaaacccctgatccaagaaatt
1601 aagaaagaaaatgagccagtgaaaaattctactcaagaaccacaagaaaa
1651 ctcaaaggtagaagagcctgaggtggcaaaagtagaagaagttgaggatt
1701 caaaaataagcgaaaataagaagccaataatagaagaggtaaaagaatca
1751 aaaatcgatgaaattccagaatcttcctctccagatacccttcccagtaa
1801 cacaggtatgttgcttatttcttcatttttttatatcttttagaagccaaa
1851 gaagttgcaagcactcccaatgaacatgattcagggtcagaggcagaagg
1901 agagattgattttgacgaatgtgactaatctttgctcataatattttatc
1951 caatcctgcttataaaatccaaaaaagggtttccttaaggtttggtgata
2001 caaagactacttcaaaatctacctaggcttggctatccatctaagcatgc
2051 aaaagacatcagaatagcctaaagcattgaaagtatggtctagagcctca
2101 aaaaattatttaaggaatctattcgagctcaggttagcggggttttgggg
2151 ttttggggttttgggg
```

B

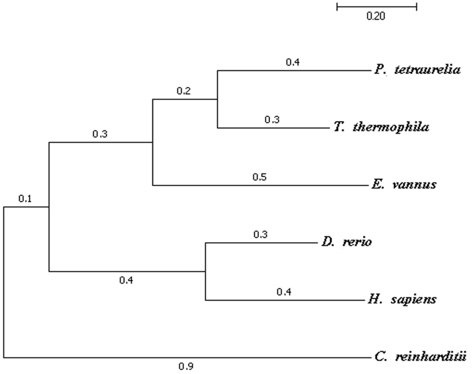

C

wt	ATG	AAC	GAC	AAT	TTA	GGT	GTA	GAC	GAG	ATA	ATT	ACC
	M	N	D	N	L	G	V	D	E	I	I	T
non-target	ATG	AAT	GAT	AAC	TTG	GGA	GTT	GAT	GAG	ATA	ATT	ACC
	M	N	D	N	L	G	V	D	E	I	I	T

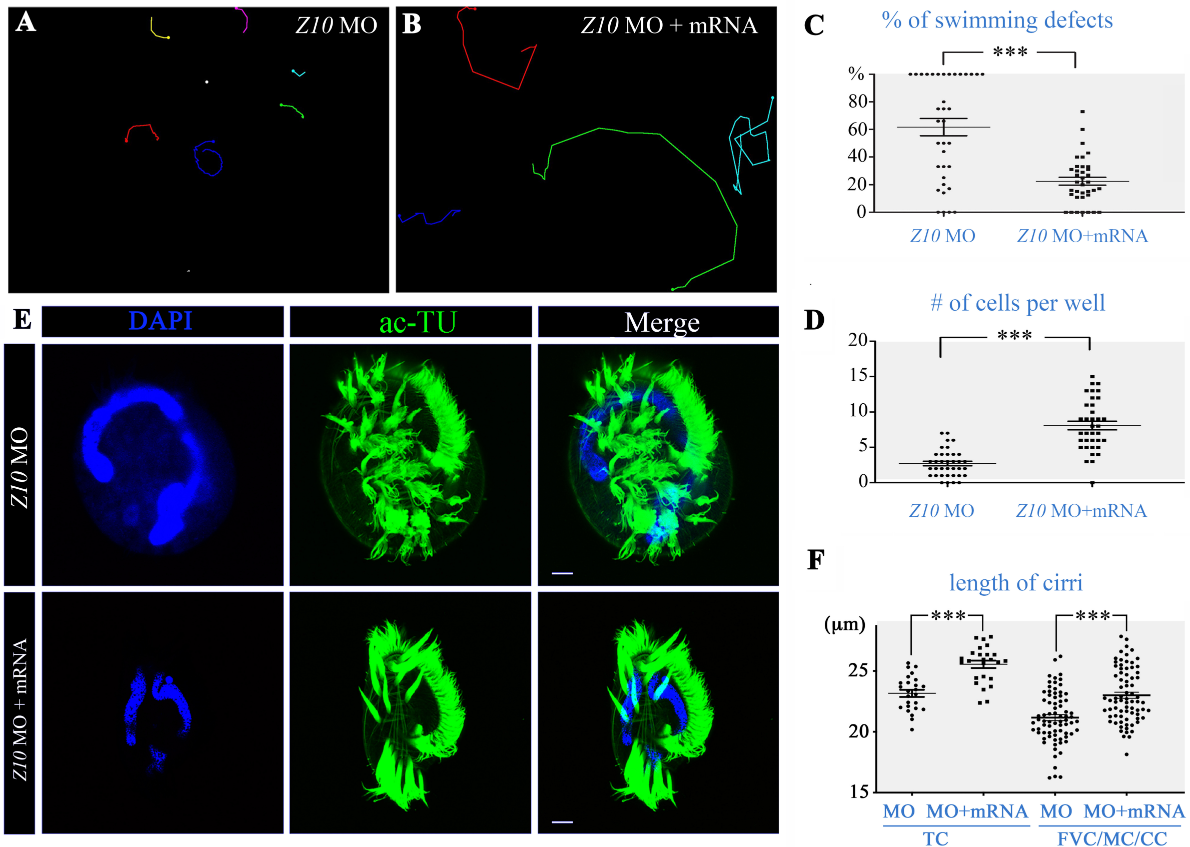

A
Z10 MO
B
Z10 MO + mRNA
C
% of swimming defects
***
%
80
60
40
20
0
Z10 MO
Z10 MO+mRNA
D
# of cells per well
***
20
15
10
5
0
Z10 MO
Z10 MO+mRNA
E
DAPI
ac-TU
Merge
Z10 MO
Z10 MO + mRNA
F
length of cirri
(μm)
***
***
25
20
15
MO
MO+mRNA
MO
MO+mRNA
TC
FVC/MC/CC

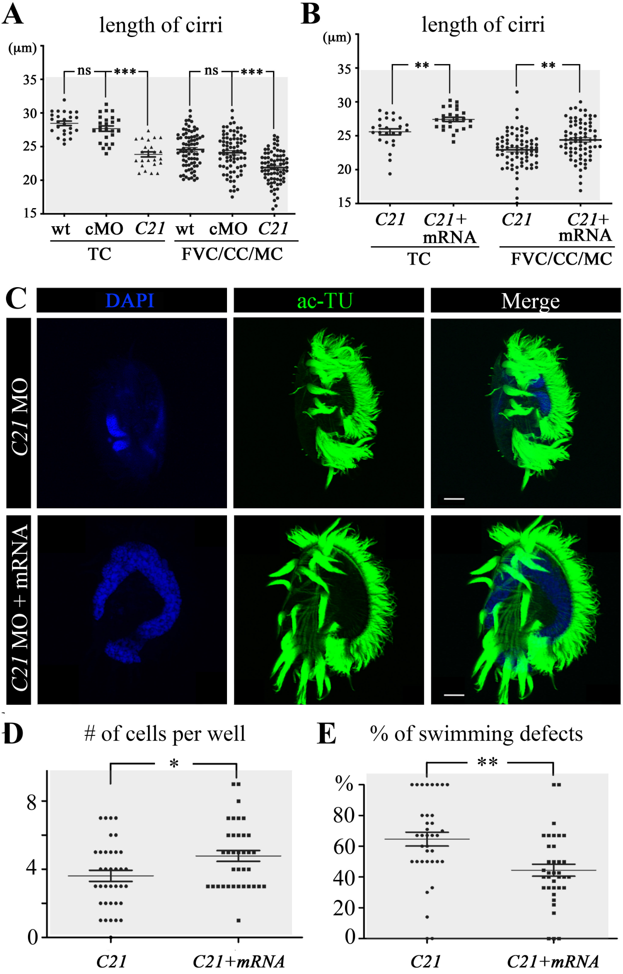

A
length of cirri
(μm)
35
30
25
20
15
ns
***
ns
***
wt
cMO
C21
wt
cMO
C21
TC
FVC/CC/MC
B
length of cirri
(μm)
35
30
25
20
15
**
**
C21
C21+ mRNA
C21
C21+ mRNA
TC
FVC/CC/MC
C
DAPI
ac-TU
Merge
C21 MO
C21 MO + mRNA
D
# of cells per well
*
8
4
0
C21
C21+mRNA
E
% of swimming defects
**
%
80
60
40
20
0
C21
C21+mRNA

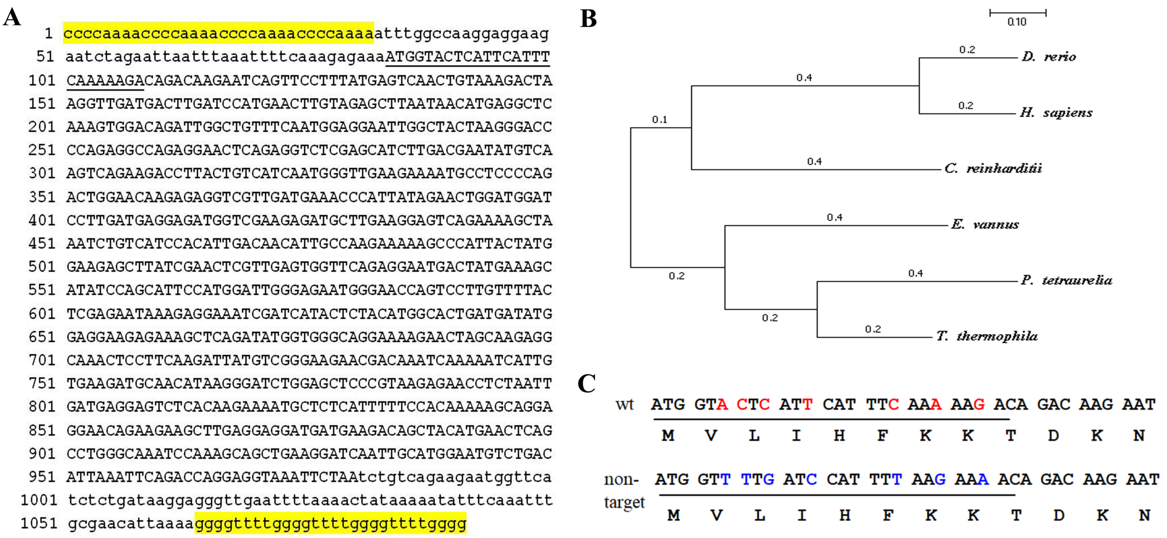
A
1 ccccaaaaccccaaaaccccaaaaccccaaaaatttggccaaggaggaag
51 aatctagaattaatttaaattttcaaagagaaaATGGTACTCATTCATTT
101 CAAAAAGACAGACAAGAATCAGTTCCTTTATGAGTCAACTGTAAAGACTA
151 AGGTTGATGACTTGATCCATGAACTTGTAGAGCTTAATAACATGAGGCTC
201 AAAGTGGACAGATTGGCTGTTTCAATGGAGGAATTGGCTACTAAGGGACC
251 CCAGAGGCCAGAGGAACTCAGAGGTCTCGAGCATCTTGACGAATATGTCA
301 AGTCAGAAGACCTTACTGTCATCAATGGGTTGAAGAAAATGCCTCCCCAG
351 ACTGGAACAAGAGAGGTCGTTGATGAAACCCATTATAGAACTGGATGGAT
401 CCTTGATGAGGAGATGGTCGAAGAGATGCTTGAAGGAGTCAGAAAAGCTA
451 AATCTGTCATCCACATTGACAACATTGCCAAGAAAAAGCCCATTACTATG
501 GAAGAGCTTATCGAACTCGTTGAGTGGTTCAGAGGAATGACTATGAAAGC
551 ATATCCAGCATTCCATGGATTGGGAGAATGGGAACCAGTCCTTGTTTTAC
601 TCGAGAATAAAGAGGAAATCGATCATACTCTACATGGCACTGATGATATG
651 GAGGAAGAGAAAGCTCAGATATGGTGGGCAGGAAAAGAACTAGCAAGAGG
701 CAAACTCCTTCAAGATTATGTCGGGAAGAACGACAAATCAAAAATCATTG
751 TGAAGATGCAACATAAGGGATCTGGAGCTCCCGTAAGAGAACCTCTAATT
801 GATGAGGAGTCTCACAAGAAAATGCTCTCATTTTTCCACAAAAAGCAGGA
851 GGAACAGAAGAAGCTTGAGGAGGATGATGAAGACAGCTACATGAACTCAG
901 CCTGGGCAAATCCAAAGCAGCTGAAGGATCAATTGCATGGAATGTCTGAC
951 ATTAAATTCAGACCAGGAGGTAAATTCTAAtctgtcagaagaatggttca
1001 tctctgataaggagggttgaattttaaaactataaaaatatttcaaattt
1051 gcgaacattaaaaggggttttggggttttggggttttgggg
B
0.10
0.2
D. rerio
0.4
0.2
H. sapiens
0.1
0.4
C. reinharditii
0.4
E. vannus
0.2
0.4
P. tetraurelia
0.2
0.2
T. thermophila
C
wt ATG GTA CTC ATT CAT TTC AAA AAG ACA GAC AAG AAT
M V L I H F K K T D K N
non-target ATG GTT TTG ATC CAT TTT AAG AAA ACA GAC AAG AAT
M V L I H F K K T D K N

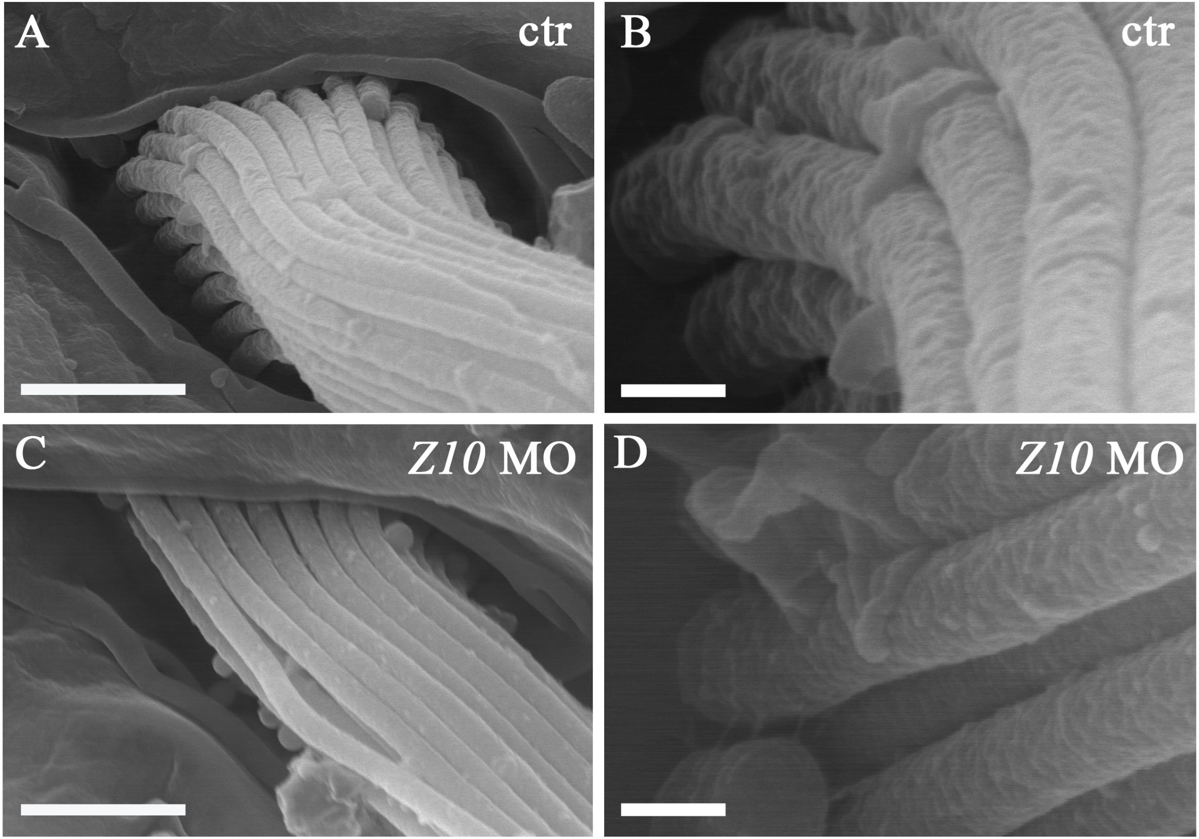
A
ctr
B
ctr
C
Z10 MO
D
Z10 MO
