# Supplementary material for: Morpholino-Mediated Knockdown of Ciliary Genes in Euplotes vannus, a Novel Marine Ciliated Model Organism
Source: Front Microbiol. 2020 Oct 19;11:549781. doi: 10.3389/fmicb.2020.549781 (PMC7604394; doi:10.3389/fmicb.2020.549781)
Supplement: Supplementary Table 1 — List of primers used in this study. [file Table_1.DOCX]

| **Table S1. List of primers used in this study** | | |
| --- | --- | --- |
| **Primer** | **Sequence（5’→3’）** | **Note** |
| ZMYND10-F1 | ggACTAGTGAGAAAATGAACAACCAAGGC | For construction of interference vector |
| ZMYND10-R1 | tccCCGCGGTTCGATGACACTATCCTGGCA |  |
| ZMYND10-F2 | ggACTAGTTCCCATGTATCCTCATTCCTC | For construction of interference vector |
| ZMYND10-R2 | tccCCGCGGGGGATTTGAGGATGCAATGGT |  |
| C21ORF59-F1 | ggACTAGTGTTGAAGAAAATGCCTCCCCA | For construction of interference vector |
| C21ORF59-R1 | tccCCGCGGGTTCCCATTCTCCCAATCCAT |  |
| C21ORF59-F2 | ggACTAGTGATCATACTCTACATGGCACT | For construction of interference vector |
| C21ORF59-R2 | tccCCGCGGTCTTCATCATCCTCCTCAAGC |  |
| Ev-ZMYND10-qF | TGCCAGGATAGTGTCATCGAA | For Real-Time PCR of *E.vannus* |
| Ev-ZMYND10-qR | GAGGAATGAGGATACATGGGA |  |
| Ev-C21ORF59-qF | GTTGAAGAAAATGCCTCCCCA | For Real-Time PCR of *E.vannus* |
| Ev-C21ORF59-qR | TTCCTCCATATCATCAGTGCC |  |
| E.coli-Z10(2)-qF | GTTCCCATGTATCCTCATTCCTC | For Real-Time PCR of *E.coli* |
| E.coli-Z10(2)-qR | TCTTTCTTGAGTTGCCACTTCTG |  |
| recA-qF | CGGGTAACCTGAAGCAGTCCAAC | For Real-Time PCR of *E.coli* |
| recA-qR | CCTGACCGATCTTCTCACCTTTGT |  |
| 18srRNA-qF | TTTTATTATCAGGGGAGGCA | For Real-Time PCR of *E.vannus* |
| 18srRNA-qR | ACCCCCAGAACCCAAAAGAC |  |
